# False-positive results released by direct-to-consumer genetic tests highlight the importance of clinical confirmation testing for appropriate patient care

**DOI:** 10.1038/gim.2018.38

**Published:** 2018-03-22

**Authors:** Stephany Tandy-Connor, Jenna Guiltinan, Kate Krempely, Holly LaDuca, Patrick Reineke, Stephanie Gutierrez, Phillip Gray, Brigette Tippin Davis

**Affiliations:** Ambry Genetics, Aliso Viejo, USA California

**Keywords:** classification discrepancy, clinical confirmation direct-to-consumer, false positive, raw data

## Abstract

**Purpose:**

There is increasing demand from the public for direct-to-consumer (DTC) genetic tests, and the US Food and Drug Administration limits the type of health-related claims DTC tests can market. Some DTC companies provide raw genotyping data to customers if requested, and these raw data may include variants occurring in genes recommended by the American College of Medical Genetics and Genomics to be reported as incidental/secondary findings. The purpose of this study was to review the outcome of requests for clinical confirmation of DTC results that were received by our laboratory and to analyze variant classification concordance.

**Methods:**

We identified 49 patient samples received for further testing that had previously identified genetic variants reported in DTC raw data. For each case identified, information pertaining to the outcome of clinical confirmation testing as well as classification of the DTC variant was collected and analyzed.

**Results:**

Our analyses indicated that 40% of variants in a variety of genes reported in DTC raw data were false positives. In addition, some variants designated with the “increased risk” classification in DTC raw data or by a third-party interpretation service were classified as benign at Ambry Genetics as well as several other clinical laboratories, and are noted to be common variants in publicly available population frequency databases.

**Conclusion:**

Our results demonstrate the importance of confirming DTC raw data variants in a clinical laboratory that is well versed in both complex variant detection and classification.

## Introduction

Direct-to-consumer (DTC) genetic tests are advertised and sold directly to the public and offer information that may include ancestry, risks of developing certain conditions, carrier status for autosomal recessive diseases, predicted drug response, and nondisease phenotypic traits such as eye color. Owing to a growing interest in human genetics and personalized health care, there has been an increased demand for this type of testing from the public. There is a growing market for DTC genetic testing, with numerous companies (e.g., Family Tree DNA, My Heritage, 23andMe, ancestry.com) currently offering products to the public. DTC tests can provide genetic information to individuals who might otherwise never have been tested due to circumstances such as lack of a family history of disease, inaccessibility of clinical genetic testing, prohibitive cost, or poor insurance coverage. However, unlike clinical genetic tests, DTC tests are not diagnostic and offer risk information for only a limited set of conditions.

In the United States, the Food and Drug Administration (FDA) restricts DTC genetic testing companies from offering products that function as diagnostic tests.^1^ In April 2017, the FDA authorized one DTC company, 23andMe, to market genetic health risk tests for 10 specific multifactorial conditions (Parkinson disease, late-onset Alzheimer disease, celiac disease, α-1 antitrypsin deficiency, early-onset primary dystonia, factor XI deficiency, Gaucher disease type 1, glucose-6-phosphate dehydrogenase deficiency, hereditary hemochromatosis, and hereditary thrombophilia).^2^ The genetic health risk tests authorized by the FDA provide information on an individual’s risk of developing a condition. This is based on the presence or absence of a limited list of genetic variants in the sample, which are statistically enriched in affected versus healthy cohorts but not necessarily causal of the conditions because additional factors such as environment and lifestyle influence an individual’s risk. None of the genes associated with these conditions are comprehensively sequenced or analyzed in DTC tests, nor do the tests include all of the genes that have been associated with these conditions. For example, 23andMe’s genetic health risk test reports on just one variant in each of two genes linked to Parkinson disease: *LRRK2* and *GBA.* ^3^ However, there are additional known pathogenic variants in these two genes as well as additional genes clinically associated with Parkinson disease that 23andMe does not report on, such as *SNCA* and *PARK2/PARKIN.* ^4^ Therefore, the consumer is not provided with a comprehensive genetic risk assessment.

In contrast, clinical diagnostic genetic tests are ordered by a patient’s medical provider and are used to identify or rule out a specific genetic condition. One example is clinical testing for the *BRCA1* and *BRCA2* genes. If an individual has a pathogenic variant in one of these genes, it is considered diagnostic for hereditary breast and ovarian cancer syndrome, whether or not she or he has a personal diagnosis of cancer. Diagnostic tests are generally comprehensive because the full coding sequences of all genes associated with a disease are analyzed. The test results are intended to be used by a patient’s medical provider to guide disease management or surveillance.

While the FDA currently prohibits most DTC companies from offering diagnostic genetic tests, some companies provide customers their raw genotyping data if requested, which may include variants in genes associated with Mendelian diseases, including those recommended by the American College of Medical Genetics and Genomics to be reported as incidental or secondary findings in genomic testing. These genes are implicated in highly penetrant genetic disorders for which surgical or other interventions aimed at preventing or significantly reducing morbidity and mortality are available to pathogenic variant carriers.^5^ Identification of a pathogenic variant in one of these genes could be diagnostic of a medical condition with potential implications for an individual’s medical management.

The raw data are often accompanied by a disclaimer that the information is neither validated for accuracy nor intended for medical use. While DTC companies do not provide interpretation of the raw data, patients can access interpretation services through third-party companies, which may charge a fee.^6^ One recent study on such third-party companies found that several operate by querying publicly available databases, such as dbSNP, and reporting the classification provided in the database, despite reports that the majority of classifications in some publicly available databases are incorrect.^6,7^ As a result, returned results may interpret particular single-nucleotide polymorphisms as pathogenic, even though clinical laboratories may classify the same variants as unknown significance, likely benign variants, or benign polymorphisms. In addition, they are providing information to the consumer with the assumption that variants in the raw data are true calls and not false positives. The misinterpretation and potential inaccuracy of the raw data pose substantial risks to individuals who obtain this type of information from a DTC company. For these reasons, medical providers should order confirmatory genetic testing from an experienced clinical diagnostic laboratory to guide patients’ medical care.^8,9,10^

What drives a consumer to pursue DTC genetic testing, their perceived usefulness of the final results, their understanding of how comprehensive a test may or may not have been, and the utilization of a genetic counselor or another health-care provider vary widely.^11,12^ DTC results may lead to healthy changes in lifestyle and/or diet,^13^ but could also result in unfavorable emotions, including anxiety when obtaining unexpected information and disappointment in a lack of comprehensive diagnostic analysis.^12^ Regardless of whether a health-care provider is involved with the initial ordering of a patient’s DTC genetic test, the results can lead to important health-related discussions with medical providers. With the ever-growing shortage of genetic counselors and other highly trained genetic professionals, there is concern regarding how DTC test results are interpreted and used among medical providers who often have minimal genetic training.^11^ It is therefore imperative that consumers, as well as their medical provider(s), are aware of the wide array of limitations to this type of genetic testing, especially in regard to an individual’s clinical management. Recent studies have started to evaluate pre- and post-DTC testing encounters with health-care providers including genetic counselors;^14^ however, to our knowledge, no studies have described outcomes of raw data confirmatory testing referrals to clinical diagnostic laboratories. We aimed to investigate the types of cases referred to our clinical diagnostic laboratory and evaluate the concordance of confirmatory test results for cases with variants identified in the raw data by DTC genetic testing. We also aimed to investigate whether our variant classification was in agreement with that provided by the DTC testing company or third-party interpretation service.^15^

## Materials and methods

Our internal database was queried to identify patients referred for testing at our clinical diagnostic laboratory (Ambry Genetics, Aliso Viejo, CA)^16,17^ with variants previously identified by DTC testing between January 2014 and December 2016. To identify such cases, all communications and curated clinical history information in our laboratory information management system were searched using key phrases including, but not limited to, “direct-to-consumer” and “DTC.” For each case identified (*n* = 49), the following information was collected: ordering provider type/specialty, test ordered, DTC results (gene, variant name, variant classification), disease status, and source of DTC results (e.g., copy of DTC report, a copy of a third-party interpretation service report, patient clinic note, information handwritten by clinician on the test requisition form). The data pertaining to the company providing the DTC testing or interpretation service were collected with the sole purpose of being able to resolve any testing discrepancies if necessary and not for publication purposes. DTC genetic test results, including reported variant classifications, were compared with confirmatory test results and were categorized as confirmed (patient determined to be positive for the variant) or not confirmed (patient determined to be negative for the variant).

While the testing methodologies used by DTC companies can vary, all testing at Ambry Genetics was performed by Sanger or next-generation sequencing analysis with Sanger confirmation, depending on the clinical test ordered. Sanger sequencing was performed on samples received for single-site analysis or full-gene analysis.^16,17^ Briefly, genomic DNA was amplified with gene-specific primers and bidirectionally sequenced using Big Dye Terminator version 3.1 on an ABI3730xl DNA analyzer (Applied Biosystems, Foster City, CA). Chromatogram analysis was conducted using Sequence Pilot version 4.2.1 (JSI Medical Systems, Boston, MA). Targeted next-generation sequencing was performed on samples received for multigene panels.^18,19^ Briefly, customized target-enrichment oligonucleotide libraries were designed using IDT xGen Lockdown probes (Integrated DNA Technologies, Coralville, IA). Genomic DNA was mechanically sheared to 300-bp fragments with an ultrasonicator (Covaris, Woburn, MA) and next-generation sequencing libraries were prepared according to the manufacturer’s instructions (Kapa Biosystems, Wilmington, MA). Adapter-ligated DNA was hybridized to custom IDT xGen Lockdown probes, eluted, and polymerase chain reaction–amplified. Final libraries were sequenced on either HiSeq2500 or NextSeq500 instruments generating 150-bp paired-end reads (Illumina, San Diego, CA).^20^

This study has been determined by regulatory opinion to be exempt from institutional review board review because it does not include human subjects. Sequence analysis is based on the following National Center for Biotechnology Information reference sequences: *BRCA1*—NM_007294.3, *BRCA2—*NM_000059.3, *CHEK2—*NM_007194.3, *CFTR—*NM_000492.3, *MEFV—*NM_000243.2, *TP53—*NM_0000546.4, *ATM—*NM_000051.3, *MLH1—*NM_000249.3, *COL3A1—*NM_000090.3.

## Results

### Study demographics and test order characteristics

Patient demographics and test order characteristics are shown in Table 1. In total, we identified 49 patients referred for clinical diagnostic testing with variants previously identified in the raw data from DTC genetic testing. There were a total of 26 unique variants submitted for testing including 4 located within deep intronic regions well beyond the analytical range of most clinical laboratories. Nearly all of the individuals in this study were female (91.8%), and most were unaffected with disease at the time of testing (73.5%). Slightly over half of individuals in this study were 30–49 years old (53.1%) and reported Caucasian ancestry (51.1%). While some individuals (*n* = 7) who underwent DTC genetic testing had a personal history of disease (affected), the majority (*n* = 35) did not have a personal history of disease reported to our lab by the ordering provider (unaffected). There was one case in which the clinical history was not available (unknown).

**Table 1:** Demographics of individuals undergoing DTC genetic testing

**Characteristic**	***N*(%)**
**Gender**	
Male	4 (8.2%)
Female	45 (91.8%)
**Age at testing**	
Under 20	0 (0.0%)
20–29	8 (16.3%)
30–39	12 (24.5%)
40–49	14 (28.6%)
50–59	4 (8.2%)
60–69	9 (18.3%)
70 and older	2 (4.1%)
**Ethnicity**	
Asian	1 (2.0%)
African American	1 (2.0%)
Ashkenazi Jewish	15 (30.6%)
Caucasian	25 (51.1%)
Hispanic	1 (2.0%)
Other/Unknown	6 (12.2%)
**Proband clinical history**	
Affected	12 (24.5%)
Unaffected	36 (73.5%)
No information provided	1 (2.0%)
**Ordering provider**	
Primary care physician	2 (4.1%)
OB/GYN	4 (8.2%)
RN/NP	5 (10.2%)
Oncologist	10 (20.4%)
Surgeon	2 (4.1%)
MD geneticist/genetic counselor	20 (40.8%)
Other	6 (12.2%)
**Test(s) ordered**	
Single-site analysis (SSA)	22 (44.9%)
Single-gene test (SGT)	13 (26.5%)
Multigene panel testing (MGPT)	10 (20.4%)
Combination of SSA/SGT/MGPT	4 (8.2%)
**Disease gene analyzed**	
Cancer	43 (87.8%)
Connective-tissue disorder	1 (2.0%)
Cystic fibrosis	4 (8.2%)
Familial Mediterranean fever	1 (2.0%)
**Source of DTC results**	
DTC report/raw data provided	13 (26.5%)
Results transcribed on test requisition form or in clinic note	26 (53.1%)
Third-party data interpretation service	10 (20.4%)

DTC, direct to consumer.

The majority of the medical providers who ordered the confirmatory testing were medical geneticists/genetic counselors (40.8%) followed by oncologists (20.4%). For 44.9% of all cases, single-site analysis was ordered to confirm DTC raw data findings, and more comprehensive testing via single-gene or multigene panel testing was ordered in 55.1% of cases. Testing of cancer genes comprised 87.8% of the orders. When samples were submitted to our laboratory, the variant of interest was reported to Ambry Genetics on the test requisition form or in a clinic note 53.1% of the time, and in only 26.5% of the cases was a copy of the DTC test results (prior to the FDA regulations) or the raw data information provided. In 20.4% of cases, a copy of the third-party interpretation service report was provided. As shown in Table 1, the vast majority of variants occurred in cancer susceptibility genes (87.8%), with the remaining variants in genes causing cystic fibrosis (8.2%), familial Mediterranean fever (2.0%), and connective-tissue disorders (2.0%).

### Variant confirmation

**Figure 1:**
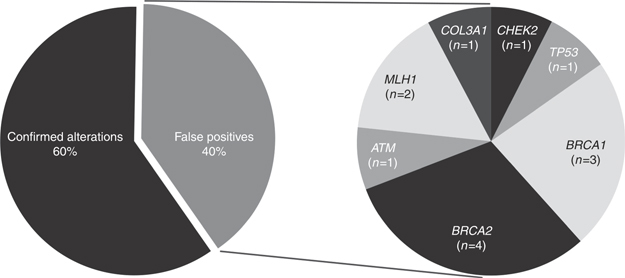
**False-positive variants in clinically actionable genes.** The pie chart on the left indicates of the variants analyzed, 60% were confirmed and 40% were false positives. The pie chart on the right shows which genes were involved with the false-positive cases and how often those false calls were detected in this study.

Overall, 60% of the variants analyzed were confirmed, while 40% were not confirmed (false positives) (Figure 1). All *CFTR* (*n* = 4, all deltaF508) and *MEFV* variants (*n* = 2) were confirmed. Of *BRCA1/2* variants identified on DTC genetic testing, pathogenic Ashkenazi Jewish founder variants were confirmed in all cases (*n* = 13), as were four additional variants; however, eight *BRCA1/2* variants yielded false-positive results. In *CHEK2*, the common 1100delC pathogenic European founder variant was confirmed in 50% of cases (*n* = 2/4) and was a false positive in the other 50% of cases. The single case reporting the *CHEK2* p.I157T founder variant was confirmed. A total of six additional variants in cancer susceptibility genes were not confirmed, including *TP53* p.R175H (*n* = 3), *ATM* p.M1040V (*n* = 1), *MLH1* p.H329P (*n* = 1), and *MLH1* c.1101delC (*n* = 1) (Figure 1,Table 2). Of the 40% of false-positive calls, 94.1% (*n* = 16/17) were in cancer-related genes and the remaining 5.9% (*n* = 1) was in a connective-tissue disorder gene.

**Table 2:** Concordance of DTC and confirmatory results from our clinical diagnostic laboratory

**Confirmed variants**	**Variant frequency**	**Ambry** ^a^	**False positives**	**Variant frequency**	**Ambry** ^a^
BRCA1 c.68_69delAG (p.E23Vfs*17)	3	PV	CHEK2 c.1100delC (p.T367Mfs*15)	2	PV
BRCA1 c.5266dupC (p.Q1756Pfs*74)	1	PV	TP53 p.R175H (c.524G>A)	3	PV
BRCA2 c.5946delT (p.S1982Rfs*22)	9	PV	BRCA1 p.E1250* (c.3748G>T)	1	PV
CHEK2 c.1100delC (p.T367Mfs*15)	2	PV	BRCA1 p.A1708E (c.5123C>A)	1	PV
CFTR p.F508del (c.1521_1523delCTT)	4	PV	BRCA1 p.R1699W (c.5095C>T)	1	PV
BRCA1 p.Q356R (c.1067A>G)	1	Benign	BRCA2 p.S1955* (c.5864C>A)	1	PV
BRCA2 p.N372H (c.1114A>C)	3	Benign	BRCA2 c.9026_9030delATCAT (p.Y3009Sfs*7)	2	PV
CHEK2 p.I157T (c.470T>C)	1	MPPV	BRCA2 p.R2336H (c.7007G>A)	1	PV
MEFV p.A744S (c.2230G>T)	1	VUS	BRCA2 c.1813dupA (p.I605Nfs*11)	1	PV
MEFV p.V726A (c.2177T>C)	1	PV	ATM p.M1040V (c.3118A>G)	1	Benign
	26 Total^b^		MLH1 p.H329P (c.986A>C)	1	PV
			MLH1 c.1101delC (p.S368Rfs*33)	1	PV
			COL3A1 p.A698T (c.2092G>A)	1	Benign
				17 Total^b^	

DTC, direct to consumer.

^a^Ambry variant classification: PV, pathogenic variant; MPPV, moderate penetrance pathogenic variant; VUS, variant of unknown significance.

^b^The combined number of variants analyzed does not equal the total number of individuals in this study (*n* = 49) because some individuals had overlapping variants in question. In addition, four variants in question were out of our reporting range and therefore not analyzed.

### Classification discrepancies

Eight variants in five genes (*ATM*, *BRCA1*, *BRCA2*, *COL3A1*, and *COL5A1*) were designated with the “increased risk” classification in DTC raw data or by a third-party interpretation service. These variants are classified as benign at Ambry as well as at several other clinical laboratories^21^ (Table 3). In addition, per the Exome Sequencing Project, 1000 Genomes, and dbSNP population frequency databases that are publicly available, these *ATM*, *BRCA1*, *BRCA2*, and *COL3A1* gene variants are found in the general population at frequencies too high to be associated with disease (Table 3) (refs.^22^,^23^,^24^).

**Table 3:** Classification discrepancies

**Gene**	**Variant**	**DTC/third party** ^a^	**Ambry** ^b^	**ClinVar** ^c^	**ESP** ^**d**^	**1000 Genomes** ^**e**^	**dbSNP** ^**f**^
*ATM*	p.M1040V (c.3118A>G)	Increased risk	Benign	Benign	1.36%	0.95%	1.48%
*BRCA1*	p.Q356R (c.1067A>G)	Increased risk	Benign	Benign	4.59%	2.81%	3.97%
*BRCA2*	p.N372H (c.1114A>C)	Increased risk	Benign	Benign	23.32%	24.26%	24.44%
*COL3A1*	p.A698T (c.2092G>A)	Increased risk	Benign	Benign	21.39%	21.16%	19.16%
*COL5A1*	c.655-8689C>T	Increased risk	Deep intronic—benign	N/A	N/A	N/A	N/A
*COL5A1*	c.654+2749A>G	Increased risk	Deep intronic—benign	N/A	N/A	N/A	N/A
*COL5A1*	c.1827+399C>T	Increased risk	Deep intronic—VUS	N/A	N/A	N/A	N/A
*COL5A1*	c.1827+1142T>C	Increased risk	Deep intronic—benign	N/A	N/A	N/A	N/A

DTC, direct to consumer; N/A, not available; VUS, variant of unknown significance.

^a^Variant classification provided by the DTC company or a third-party interpretation service.

^b^Variant classification provided by Ambry.

^c^Variant classification provided in ClinVar (clinical laboratory submissions only).

^d^Exome Sequencing Project population frequency database.

^e^1000 Genomes population frequency database.

^f^dbSNP population frequency database.

### Case example: Ehlers–Danlos syndrome

An in-depth review of the *COL3A1* case revealed that the patient was undergoing evaluation to rule out Ehlers–Danlos syndrome and presented DTC genetic testing results to the clinician revealing one *COL3A1* “mutation” and four *COL5A1* “mutations.” To confirm these findings, the clinician ordered a 22-gene panel for thoracic aortic dilation and dissection, including *COL3A1* and *COL5A1*, and received a negative report. No DTC report was provided at the time, so the precise variants in question were unknown. Upon follow-up communication with the provider to request the DTC report, we received a report from a third-party DTC raw data interpretation service that revealed the following variants for this patient: *COL3A1* p.A698T (c.2092G>A), *COL5A1* c.655-8689C>T, *COL5A1* c.654+2749A>G, *COL5A1* c.1827+399C>T, and *COL5A1* c.1827+1142T>C. The *COL3A1* variant was not detected by our lab in this patient, and it would not have been reported if it had been detected, as our laboratory classifies this as benign. Furthermore, the *COL3A1* and all four of the *COL5A1* variants were labeled as increasing the patient’s risk of disease; however, the *COL3A1* variant and three of the *COL5A1* variants were classified as benign by our laboratory, with the fourth *COL5A1* variant classified as a variant of unknown significance. In addition, all four *COL5A1* variants were located in deep intronic regions not included in our analytical range due to unproven association with disease (Table 3).

## Discussion

The recent proliferation of DTC companies increases the general population’s access to genetic testing, including healthy individuals. DTC test results are not intended to impact an individual’s medical management; however, information obtained from requesting and interpreting raw data could lead to inappropriate changes in their care. While the raw data include disclaimers stating that they have not been validated for accuracy and are therefore not intended for medical use, they could easily be misinterpreted or misused by a consumer or medical provider with little to no training on the complexities of genetics. Both false-positive results and misclassification of variants can result in significant implications for an individual, including unnecessary stress, medical procedures (e.g., surgery, frequent screenings), and testing of family members. All of these factors have the potential to result in unwarranted financial burden on individuals and the health-care system overall.

This study focused on variants reported in the raw data of DTC genetic testing and our two key findings were an alarmingly high false-positive rate (40%) and the incidence of discrepant classification/misinterpretation of variants coming from DTC companies and/or third-party interpretation services. The technical differences between the types of testing methodologies used may explain why 40% of the results in our study were discordant with the raw data from the DTC testing company. Many of the DTC genetic testing laboratories use a form of single-nucleotide polymorphism genotyping array for their assay. This particular methodology is analogous to spot checking an individual’s DNA with coverage at only specific preselected sites. This is not comprehensive full-gene sequencing nor does it include gross deletion or duplication analyses, which are both routinely part of clinical diagnostic testing with the use of next-generation sequencing and microarray/multiplex ligation-dependent probe amplification methodologies. Even when comparing single-nucleotide polymorphism arrays between DTC companies, it is possible to see a high degree of variability, as probe coverage varies between companies due to differences in assay design.

Encouraging all laboratories, whether they are DTC or clinical, to share their data is one way to reduce variant classification errors.^25,26,27^ Requiring DTC companies and any associated third-party interpretation services to utilize and review information from well-curated and highly regarded genetic databases could also aid in reducing these types of errors.^5^ These third-party reporting services typically do not use any form of multievidence algorithms or weigh various interpretation factors, such as classification discrepancies between testing laboratories, despite the availability of this information in some public databases such as ClinVar. In the meantime, it is crucial that clinical confirmatory testing be performed on any variants reported in the raw data provided by a DTC company prior to any changes in medical management to confirm the presence of that variant in the individual as well as an accurate classification.

In addition to concerns regarding false-positive results and inaccurate classification of variants as pathogenic versus benign, all parties involved with these types of test results should be aware that many DTC genetic tests do not include comprehensive gene analysis, and an individual may still need additional clinical diagnostic genetic testing based on personal and/or family history. It is important that consumers and their health-care providers are cognizant of the differences in testing methodologies between clinical diagnostic labs and DTC testing labs so that there is no false reassurance or alarm on the behalf of the individual or the health-care provider.

All of these potential ramifications highlight the need for clinical confirmation testing of raw data variants. The 49 cases represented in this study are from individuals who shared their DTC genetic test results with their medical providers, and they represent the circumstances in which the medical providers knew the results warranted follow-up clinical testing. Unfortunately, these actions on part of both the individual and the medical provider may not be commonplace at this point in time. A limitation of this study design is that we were unable to analyze the complete financial burden that these types of test results may place on our health-care system. While this study has a small sample size (*n* = 49), further efforts to gather data on a larger cohort are underway. This will allow us to better determine the extent of discordant results between DTC tests and clinical diagnostic tests. The findings in this study are also limited to the genes for which our clinical diagnostic laboratory offers testing (https://www.ambrygen.com).

## Conclusion

Our analysis revealed a high false-positive rate (40%) in genes with potential clinical impact in the raw genotyping data provided to consumers by DTC genetic testing companies, as well as eight instances of misinterpretation of variants by third-party interpretation services. This emphasizes the limitations in raw genotyping data currently distributed by DTC companies and demonstrates the importance of confirming DTC raw data results in a diagnostic laboratory that is well versed in clinical-grade variant detection and classification. While having access to raw genotyping data can be informative and empowering for patients, this type of information can also be inaccurate and misinterpreted. Genetic testing needs to be interpreted by a qualified health-care professional in the context of several other factors, such as personal and family medical history. It is our hope that confirmatory testing and appropriate clinical management by all health-care professionals accompany DTC genetic testing for at-risk patients.
